# Can construction helmets save lives? Evidence from a biomechanical reconstruction of a work-related head trauma

**DOI:** 10.1007/s00414-025-03695-9

**Published:** 2026-01-06

**Authors:** Natalia Lindgren, Svein Kleiven, Xiaogai Li

**Affiliations:** Division of Neuronic Engineering, KTH Royal Institute of Technology, Hälsovägen 11C, Huddinge, 141 57 Sweden

**Keywords:** Accident reconstruction, Injury prediction, FE head model, Skull fractures, Traumatic brain injury

## Abstract

Each year, 320 000 people die from occupational injuries. The construction sector is one of the most hazardous sectors, showing a high incidence of workplace fatalities, of which many are caused by traumatic head injuries. In this study, the efficiency of construction helmets has been investigated through an in-depth accident reconstruction of a real-world workplace head trauma, aiming to investigate causation, prevention and liabilities in an ongoing police investigation. The accident was reconstructed with a state-of-the-art subject-specific head model, used to predict the skull fracture and the brain’s response to impact. The results of this study show how the skull fracture pattern was predicted with striking resemblance to the real-world fracture and how the locations of high brain strains were predicted in accordance with the victim’s brain lesions. The impact scenarios were compared with the hypothetical scenario in which a construction helmet was worn during the impact. The comparison provides evidence to support that a helmet would have prevented the skull fracture, and possibly also life-threatening brain injury. This case study demonstrates how FE reconstructions can help prove causality and liability in fatal head traumas. More importantly, the findings highlight the role of safety helmets in preventing lethal head injuries and their importance in combating the globally high incidence of fatal work-related accidents.

**Supplementary Information:**

The online version contains supplementary material available at 10.1007/s00414-025-03695-9.

## Introduction

320,000 people die from occupational injuries globally each year [1], and traumatic head injuries account for more than a fifth of fatal work-related accidents in the EU and US[2, 3]. The construction sector is one of the most hazardous sectors, showing a high incidence of workplace fatalities and fatal head injuries [2, 4–6]. Adding to the magnitude of this issue, establishing liabilities in a workplace accident has shown to be notoriously difficult [7]. In Sweden alone, where more than one person dies each week in workplace accidents [8], only a tenth of the fatal workplace accidents end up with a criminal conviction. Few of the police reports of work environment offences causing another’s death even lead to prosecutions: in 2017, two thirds of the preliminary crime investigations were closed before being passed on to prosecutors, with one of the main reasons being a lack of evidence [9].

A technique that has the potential to help in obtaining supporting evidence in accidents with a fatal or injurious outcome is Finite Element (FE) accident reconstructions. Such reconstructions involve using computational simulations to recreate and analyze an accidental event on the basis of classical mechanical laws. In short, FE involves breaking down a geometrically complex body into smaller, simplified pieces, i.e. finite elements, collectively called a mesh. By solving a system of partial differential equations, one can subsequently calculate how each element, and by extension the whole system, deforms under load [10]. By creating FE models of the human body, or parts of it, the technique can be used to scrutinize human response to impact.

FE human body models (HBMs) and FE head models are currently being used as experimental surrogates for studying human injuries across a wide variety of fields, mainly within automotive safety, but also in areas such as ergonomics and sports science [10]. Occasionally, FE models have also been used within the field of forensic medicine to help prove causality and distinguish between inflicted and accidental causes [11–15]. For example, in 2019, Li et al. [12] published FE reconstructions of two alleged child abuse cases. By using a subject-specific FE head model, the researchers could provide biomechanical evidence to support the forensic investigation of determining whether or not the infants’ traumatic head injuries were caused by domestic abuse. A few years earlier, in 2006, Kleiven [11] published an FE reconstruction of a case where a woman had sustained a lethal trauma to the head. By using FE analysis to investigate the forces that would be required to cause the woman’s documented head injuries, Kleiven could provide insights that challenge the notion of accidental causation in the investigated fatality.

Although FE was introduced in the field of forensic medicine almost 20 years ago [11], and subsequent researchers have pointed out its promising potential in routine forensic practice [13, 16, 17], very few examples of its application in the field can be found in the current body of literature. Regardless, some suggest FE modeling in forensic practice may open up for an objectification of forensic evaluations, potentially reducing the risk of diverging medical opinions sometimes seen in court cases [18]. In addition to that, FE modeling, particularly when used for in-depth reconstructions of real-world accidents, is already being widely used to target intervention strategies and help enhance our understanding of head injury mechanisms [10]. The latter is inevitably needed when it comes to combating work-related head injuries, particularly within the construction sector.

The objective of the current study is to further exemplify how biomechanical FE reconstructions can be used to prove causation and liabilities in cases of fatal head traumas. This study will also be aimed at evaluating the ability of construction helmets to prevent skull fractures and brain injuries. In pursuit of these aims, the current article is framed around a selected case of a real-world workplace fatality, in which a construction worker suffered lethal head injuries following trauma to the head at a construction site in Sweden. Since the victim was not wearing a helmet at the moment of impact, and was thus not taking the mandatory safety precautions, the question of liability arose during the police investigation: could the employer be held liable for criminal negligence causing death, while the victim failed to comply with the workplace safety regulations?

The current study endeavors to reconstruct the referred-to workplace accident numerically using state-of-the-art reconstruction technologies, in order to analyze the significance of the helmet in the impact situation. Understanding the role of a helmet in the injury outcome of this case might help to answer the arisen question of liability. The focus of the study is to first identify a plausible accident scenario, to thereafter compare it with the hypothetical scenario in which the victim had been wearing a helmet. The victim’s traumatic head injuries will be predicted using subject-specific FE head models and FE human body models (HBMs).

## Case description

An outline of the workplace accident was established based on the preliminary police investigation reports. The reports included police and witness statements, photographs taken from the accident scene, as well as the victim’s medical journal. The victim’s medical images were provided by the police, including computed tomography (CT) and magnetic resonance imaging (MRI) scans of the victim post-accident.

**Fig. 1:**
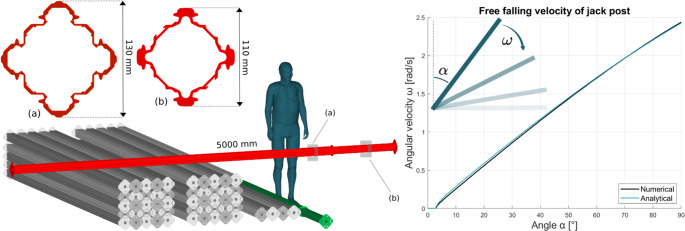
Accident scene and impact velocity of jack post. Left: A sketch of the accident scene: according to witness statements, the victim was occupied with adjusting an extended stamp (seen in green). After the accident, the falling stamp (seen in red) was found on top of the jack post piles. A cross-section view of the lower and upper part of the impacting jack post is provided, see view (a) and (b) respectively, along with its dimensions. Right: The derived angular velocity of the free falling jack post as a function of the incline angle of the jack post

A sketch of the accident scene was constructed based on the acquired material and is provided in Fig. 1. According to witness statements, the victim was positioned next to a pile of jack posts (adjustable metal poles often used for temporary structural support) at the time of the accident. A second witness states that the victim was positioned “hunched over” one of the laying jack posts. The victim was reportedly adjusting the laying jack post as an upright standing jack post fell, striking the victim on the crown of his head. The falling jack post was later found on top of the jack post piles by the police. The falling jack post was made of aluminum and had a weight of 34.8 kg and length of 5 m, with the geometry and dimensions shown in Fig. 1.

**Fig. 2:**
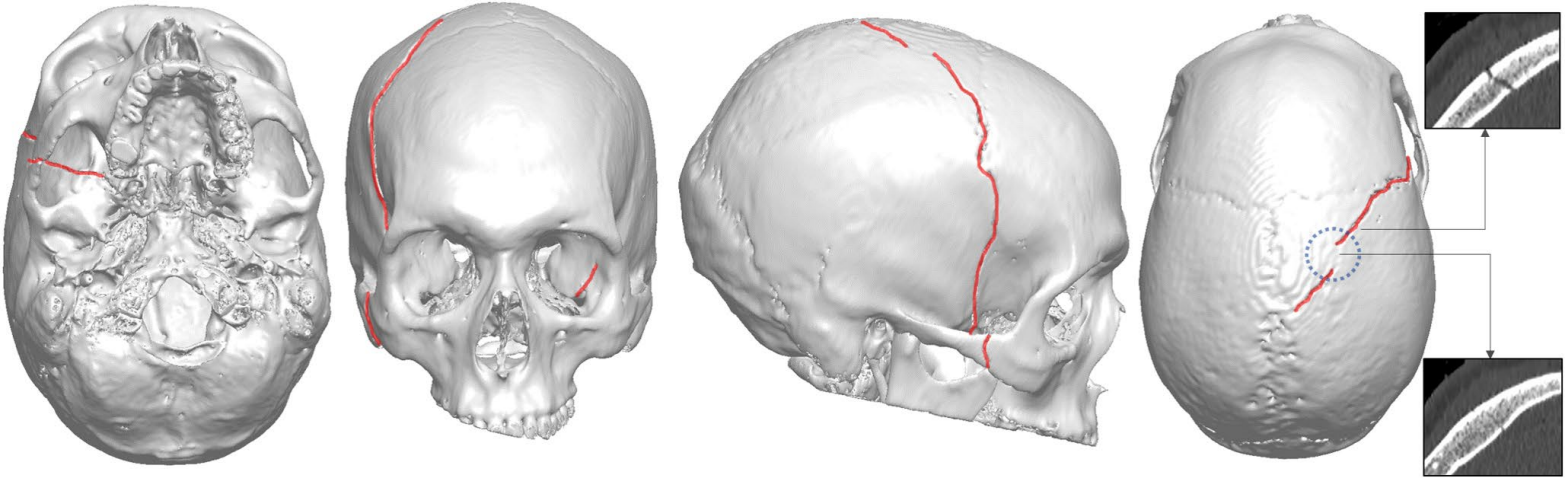
3D visualization of the victim’s skull bone seen from below, front, right and above. The fracture, highlighted in red, goes from the top of the head in the parietal bone towards the right side and continues along the skull base to the left sphenoidal sinus. Note the fracture on the right zygomatic bone and the interruption in the fracture, emphasized by the CT cross sections at two locations. The estimated region of impact is highlighted by the blue dotted circle on the top view of the cranium

The victim sustained a right-sided skull fracture and smaller intracranial bleedings, as well as a general edema within the brain parenchyma and brain contusions. Subarachnoid bleedings were found bilaterally. A smaller subdural hematoma was found on the left side, along with a minimally thin subdural hematoma close to the skull fracture to the right. The fracture extended from the top of the head in the parietal bone to the right side of the cranium, following a widened suture. The fracture line continued from os sphenoidale in the frontal skull base to the left-side sphenoidal sinus that was filled with hematoma. Figure 2 shows the segmented cranium with the visualized fracture. The segmentation was made based on the victim’s post-accident CT using thresholding tools. Note the small fracture interruption on the crown of the head, where the fracture did not go through the whole skull thickness. Unfortunately, the victim did not survive and was pronounced dead a few days later.

## Materials and methods

The victim of the aforementioned workplace accident sustained a skull fracture and various injuries to the brain. These injury types are associated with different injury mechanisms and have therefore been studied separately in this study – see a detailed motivation behind this methodological choice in Supplementary Appendix D.

**Fig. 3:**
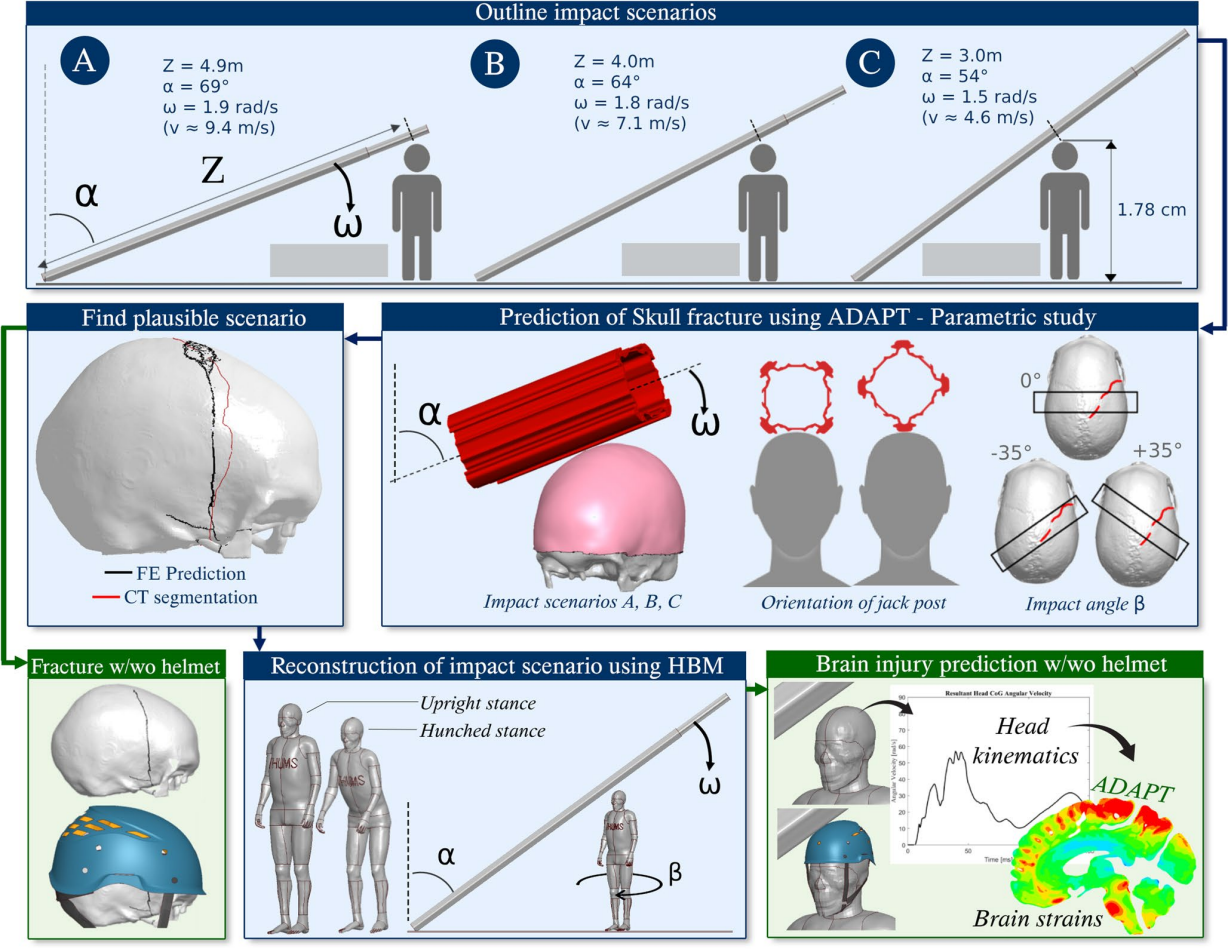
Study overview. Firstly, three plausible impact scenarios (Scenario A, B and C) were outlined for further investigation. Secondly, a parametric study was performed using the subject-specific head model (ADAPT) until the fracture was predicted in agreement with the victim’s medical images. Third, the head kinematics and brain deformations generated by the impact was studied with whole-body models (THUMS) positioned with two different postures. The final impact scenario was reconstructed both with and without a worn helmet for a comparative analysis

A schematic overview of the study approach is provided in Fig. 3. In the following sections, the reconstruction procedure will be described in detail starting with a description of the current accident case and any made inferences, followed by a presentation of the skull fracture and brain injury prediction approach. All referred-to reconstructions were preprocessed in LS-PrePost v4.8 and simulated in LS-DYNA v13 using multiple CPUs. Postprocessing was done in MATLAB v2021b. 3D Slicer v4.11 (open-source software available at www.slicer.org) was used for segmentation of medical images.

### Impact point

Linear skull fractures, i.e. skull fractures that follow a line and are not splintered or depressed, have for long been hypothesized to emerge due to tensile stresses inherited from the outwards bending of the cranium at a distance away from the impact region [19]. Because of this outward bending, a linear fracture usually extends towards the impact point, as well as away from the impact point, towards areas that are structurally weaker, such as the skull base [20]. In experimental studies, researchers have also observed how microfractures in the skull bone’s inner cortical layer occur right below the impact point, before fracture is initiated away from the impact point and propagated towards it[17, 21]. With this fundamental knowledge about skull fracture mechanisms, an estimation was made regarding the impact location of the falling jack post. The impact location, highlighted in Fig. 2, was assumed to be in the region of the “interruption” of the fracture on the crown of the victim’s head.

### Accident scenarios

Before impacting the victim’s head, the jack post was assumed to have fallen freely from upright position. The jack post’s free falling velocity was calculated using FE analysis. A meshed jack post, 5 m long and 34.8 kg heavy, was positioned upright with an initial incline of $\:{\alpha\:}_{0}$ = 3°. The jack post was allowed to fall freely with only gravity as load. Translational constraints were applied at the bottom node to create an axis of rotation. The resulting angular velocity of the jack post is included in Fig. 1. The angular velocity can also be derived analytically by applying conservation of energy. By expressing the potential energy at an arbitrary angle 𝛼 and relating it to the rotational kinetic energy, the following function can be derived:

1 $$\:\omega\:\left(\alpha\:\right)=\sqrt{\frac{3g}{L}\left(\mathrm{cos}{\alpha\:}_{0}-\mathrm{cos}\alpha\:\right)}$$

with *L* denoting the length of the falling beam. The analytical function is included in Fig. 1 and aligns well with the simulation results.

It is not known how far away the victim was standing from the falling jack post, as no measurements were taking from the scene. The translational impact velocity would consequently differ depending on the victim’s position relative to the jack post’s point of rotation. Three hypothetical impact scenarios, presented in Fig. 3, were evaluated in this study. Scenario A would translate as the most severe case, where the victim would be standing at a distance from the jack post so that the top of the post would hit the head with a resultant velocity of about 9.4 m/s. In Scenario B and C, the victim would be placed so that the resultant impact velocity would be approximately 7.1 and 4.6 m/s respectively. All three scenarios are associated with different impact rotational velocities 𝜔 and with different inclines 𝛼 of the jack post. Note that due to the design of the jack post (Fig. 1), the cross-section geometry of the impacting part of the jack post differ in Scenario A compared to Scenario B and C.

It is not known how the victim fell after impact and if any secondary head impacts occurred. All head injuries were assumed to be associated with the initial jack post impact, and potential ground impacts were not included in the analysis.

### Predicting skull fracture

**Fig. 4:**
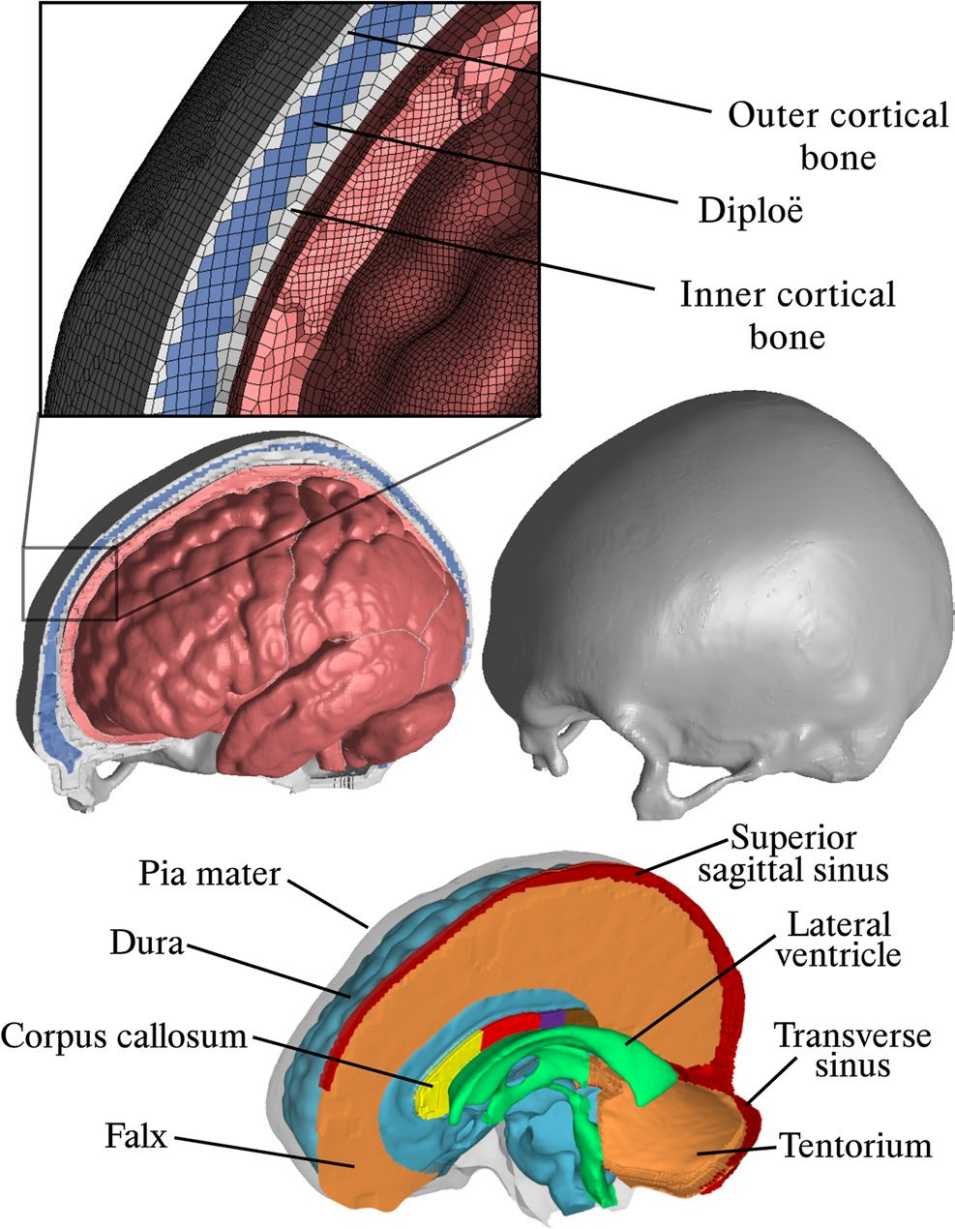
The ADAPT head model and its constituents

To reconstruct and investigate the victim’s skull fracture in-depth, the ADAPT model [22, 23] was used. The ADAPT model, visualized in Fig. 4, is an anatomically detailed FE head model that includes the brain and several of its constituents, as well as an enclosing cranium. The elements of the ADAPT model are between 0.2 and 0.5 mm in size, a resolution high enough to consider fracture propagation.

The human skull can be regarded as a three-layered structure, with two layers of compact cortical bone covering the outer and inner layer of the skull, and a more porous bone layer in-between referred to as trabecular bone or diploë. This bone structure, which is very significant for the mechanical behavior of the cranium, is included in the ADAPT model. The cranial model, defined with a strain-rate dependent material, has previously been shown to be successful in predicting skull fractures in fall accidents [17].

By using morphing techniques [13, 22], the ADAPT geometry can be individualized to match a subject of interest. The CT images of the victim’s head were used to reshape (morph) the ADAPT model to have the same morphology as the victim. Using this type of subject-specific models is highly important for fracture prediction, as the skull morphology and thickness has shown to be very influential for fracture propagation [17, 24].

The scalp thickness was measured to be 6 mm at the derived region of impact, observed in the CT scans. Thus, a 6 mm thick scalp (modeled with six layers of solid elements) was applied to the ADAPT model. The scalp was modeled with an exterior layer of dermis and an inner layer representing the two adipose layers of the scalp and the intermediated galea aponeurotica [17]. Since the ADAPT model does not include any facial structures, an extra weight of 0.6 kg as added to the skull base, leading to a total head mass of 4.0 kg, which is in better agreement with earlier reports on human head mass [25].

The jack post was meshed and then placed above the subject-specific ADAPT model with different impact angles 𝛽, in all instances targeting the initial contact at the derived impact region. The impact angle, denoted 𝛽, corresponds to different orientations of the victim relative to the jack post, see Fig. 3.

### Predicting brain injury

To study the head kinematics during and after impact, an HBM was used to reconstruct the event. The HBM used in this study was the Total HUman Model for Safety (THUMS) [26], an HBM distributed by Toyota. THUMS is aimed to represent an average sized male (178.6 cm, 77. kg) and is widely used within the field of impact biomechanics as well as the vehicle industry. The HBM is anatomically detailed with skeleton, organs, muscles and brain, however with a significantly larger element size (element size between 3 and 5 mm) in comparison to the ADAPT model.

Two postures that were brought up by witnesses were investigated. The two postures represent scenarios where the victim was standing upright and where the victim was positioned standing leaning forward, in a slightly hunched posture, see Fig. 3.

After the accident had been reconstructed using the HBM, the kinematics pulses (the angular velocity and acceleration of the HBM’s head center of gravity) were applied to the ADAPT model and the strains of the brain tissue were studied.

### Helmet

Once a plausible accident scenario was found, the same impact configuration was also simulated with a worn helmet. This way, a comparison of the injury outcome with and without wearing a helmet could be made. The helmet represents a construction helmet currently available in the Swedish market, equipped with an outer shell of ABS and an inner lining of EPS foam. It includes an adjustable strap for the head and chin, as well as a Mips low friction layer (LFL). The helmet model is validated against standard impact tests EN397 and EN12492s, involving drop impacts at different heights against the crown on the head, see Supplementary Appendix A for further details. The impacts were reconstructed with the worn helmet both with and without the LFL.

### Injury prediction metrics

Based on the victim’s medical reports, the CT and MRI revealed the following types of head- and brain injuries: skull fracture, subdural hemorrhages (SDH), subarachnoid hemorrhages (SAH), contusions and edema. Several experimental studies using post mortem human subjects (PMHS) have been published over the decades, investigating appropriate metrics and thresholds of some of these injuries. However, human tolerance thresholds, meaning, the biomechanical limits of the human body beyond which injury is likely to occur, is a subject under debate. A selection of relevant experimental findings regarding human injury thresholds are presented in Table 1. Note that the listed thresholds are not definite and consensus on human tolerance levels has not been reached.

**Table 1:** Summary of used injury thresholds and risks in terms of peak fracture forces, peak linear accelerations (PLA), angular accelerations (PAA), angular velocities (PAV) and maximum principal strains (MPS)

Injury	Authors	Description of study	Threshold value
Skull fracture	Allsop et al. [27]	Reported peak fracture forces in rectangular plate impacts. 11	5800–17,000 N
		PMHS head specimens and temporo-parietal impacts.	
	Delye et al. [28]	Reported peak fracture forces in double-pendulum impacts	5940–14,300 N
		with flat surfaced impactor. 18 PMHS head specimens and	
		frontal impacts	
	Monea [29]	Reported peak fracture forces in double-pendulum impacts	4360–14,300 N
		with flat surfaced impactor. Impact velocities > 4 m/s. Occip-	
		ital, frontal and temporal impacts.	
	Hodgson et al. [30]	Reported peak fracture forces in guided drop of test of cylin-	4225–7340 N
		drical surface (⌀5 cm) against stationary head. 12 PMHS head specimens, frontal impacts	
	Yoganandan et al. [31]	Reported peak fracture forces in impacts with piston on sta-	4640–14,000 N
		tionary heads. 12 specimens, different skull impact regions	
	Yoganandan et al. [32]	Reported peak fracture forces in lateral drop impacts against	5560–9920 N
		rubber flooring. Four specimens, lateral impacts	
	Mertz et al. 1997	Dummy experiments	5% risk if PLA > 180 g
SDH	Löwenhielm [33]	Experimental tests on PMHS, carseat sled impacts	PAA > 4.5 krad/𝑠^2^
	Löwenhielm [33] Newman [34] Depreitere et al. [35]	Experimental tests on PMHS, carseat sled impacts Review of reported head injury data Head impact tests on PMHS	PAV > 50 rad/s PAA > 10 krad/𝑠^2^ PAA > 10 krad/𝑠^2^
	Lee & Haut [36]	Axial stretching of parasagittal bridging veins, eight PMHS	MPS > 0.53
	Monson [37]	Dynamic stretching of cerebral blood vessels, 25 subjects	MPS > 0.50
	Delye et al. [38]	Dynamic stretching of parasagittal bridging veins, nine PMHS	MPS > 0.25
DAI	Bain & Meaney [39]	Experimental stretching of the right optic nerve of adult male	Brain white matter dam-
		guinea pigs	age at MPS > 0.21
	Morrison et al. [40]	Studied thresholds for cell death using cultures of brain cells	Cell death at MPS > 0.20
	Thibault et al. [41]	Uniaxial tension experiments of giant squid axons	Structural failure of ax-
			ons when MPS > 0.25
	Margulies & Thibault [42]	Experiments for the human head subjected to lateral motion	Moderate to severe DAI when PAA > 16 krad/𝑠^2^
Concussion	Fahlstedt et al. [43]	Reconstructions of 53 head impacts of American football	50% risk of concussion
		players	when brain MPS > 0.28

Skull fractures are categorized as a contact induced injury and they occur when the applied contact load reaches a magnitude that cause the skull to break. Thus, as seen in Table 1, skull fractures are associated with metrics in terms of forces and linear accelerations.

Brain hemorrhages are accumulated blood within the brain as a result of tearing of veins or arteries. In SDHs, the bleeding is found between the dura and arachnoid meninges. In SAHs, the bleeding is accumulated in the subarachnoid space. SDHs, which are one of the most lethal of head injuries, are believed to occur due to rapid and high rotational accelerations. The most common mechanism of SDH is ruptured veins that bridge the brain surface and intracranial subdural space [44, 45]. It has been thus been suggested that SDH is produced by short duration and high amplitudes of angular acceleration, see Table 1. To the authors knowledge, no thresholds specific for SAH have been reported.

Diffuse Axonal Injury (DAI) are characterized by wide-spread damage of the white matter of the cerebral hemispheres. DAI occurs due to rotational and/or rapid accelerations of the head causing damage to many of the brain’s axons. DAI is one of the most severe brain injuries, where a majority of patients with severe DAI never regain consciousness [46, 47]. There were no obvious signs of DAI according to the medical reports. Experimental findings relating to the human tolerance levels for DAI are presented in Table 1.

Brain contusions are generally found at the site of impact or the opposite side, so-called coup contrecoup injury. In general, contusions are believed to originate from straining of brain tissue that occur due to rapid head rotations [48, 49]. Brain contusions occur have been attributed to bleeding from the continuous flow of injured microvessels that ruptured during impact [50]. If a skull fracture is present, however, contusions are believed to occur due to the skull pressing against the underlying brain tissue [45, 48].

## Results

The three impact scenarios (A, B and C), were reconstructed using the ADAPT head model, impacting the HBM straight from the right, i.e. impact angle 𝛽 = 0°. A skull fracture was predicted by the model in all three scenarios. Scenario A and B resulted in severe skull fractures, where the model predicted a depressed and comminuted fracture. In Scenario C, a linear skull fracture was predicted by the model, following a path from the impact point towards the frontal lobe. Due to the severity of the fractures predicted in Scenario A and B, these two scenarios were omitted as non-plausible while Scenario C, with an incline angle 𝛼 = 54° and angular velocity of 𝜔 = 1.5 rad/s ($\:{v}_{res}$ ≈ 4.6 m/s), was evaluated further. The predicted fractures of Scenario A and B are provided in Supplementary Appendix B.

**Fig. 5:**
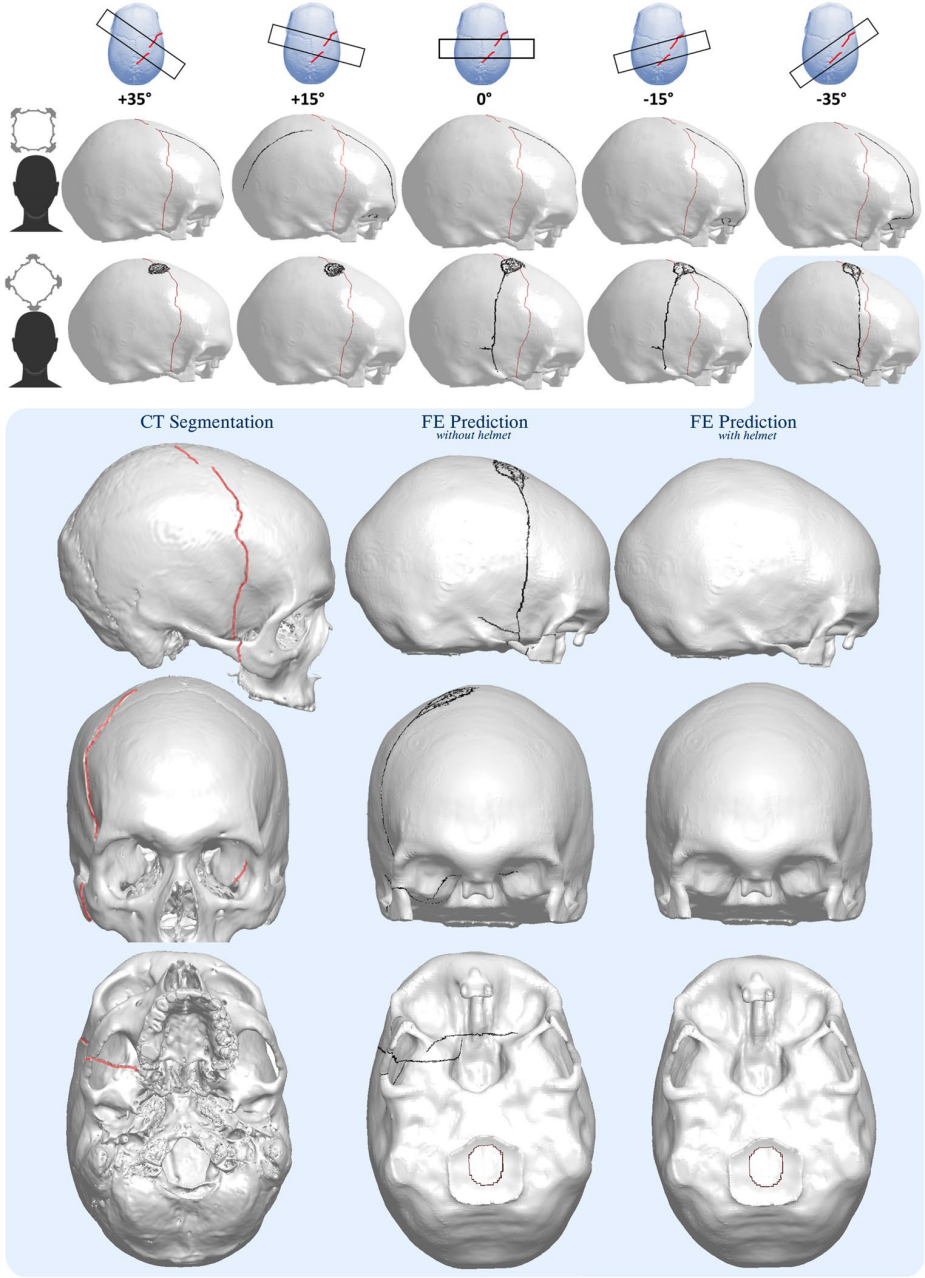
Predicted skull fractures in a selection of impact modes, altering the impact angle of the jack post and its impacting surface. The red fracture line represents the fracture segmented from CT, while the black represents the through-thickness-fracture predicted by the FE model. The impact scenario that predicted the fracture similar to the fracture of the victim is presented in detail

Continuing the investigating of Scenario C, a range of impact angles (− 55° < 𝛽 < +55°) were studied, some of them presented in Fig. 5. The axial rotation of the jack post was also altered, either impacting the head with the post’s “flat” side, or with one of its “corners”. As a result, only one impact mode was found that resulted in a skull fracture similar to the victim’s. When the “corner” of the jack post impacted the head with a −35° impact angle, a linear fracture was predicted, extending from the parietal bone to the skull base, almost all the way to the left orbital roof. A fracture of the zygomatic bone was also predicted in agreement with the victim’s fracture. The fracture was however not predicted with a fracture “interruption” and extension towards the crown of the parietal bone. The predicted fracture is presented in detail in Fig. 5. This impact scenario, deemed the most plausible according to the prediction results, was also simulated with a worn helmet (without LFL). With a worn helmet, no skull fracture was initiated according to the FE prediction, as illustrated in Fig. 5.

From the CT segmentation, a swelling was observed near the assumed region of impact. It was found that these regions of swelling aligned well with the pattern of scalp deformation predicted by the ADAPT model. The scalp’s deformation during the simulated impact is presented in Supplementary Appendix B, along with a thickness map of the scalp, which was generated from the segmentation of the victim’s CT.

Ultimately, a plausible impact scenario was found that could predict the victim’s skull fracture: an incline angle 𝛼 = 54°, an angular velocity of 𝜔 = 1.5 rad/s ($\:{v}_{res}$ ≈ 4.6 m/s) and an impact angle of 𝛽 = −35°. This particular scenario was simulated using the HBM to extract the head kinematics. Since the victim’s posture prior to impact was unknown, two cases were considered- one scenario where the victim was standing upright, and one where the victim was leaning forward, slightly hunching. Note that the same head impact location was targeted in the two postured HBMs.

**Fig. 6:**
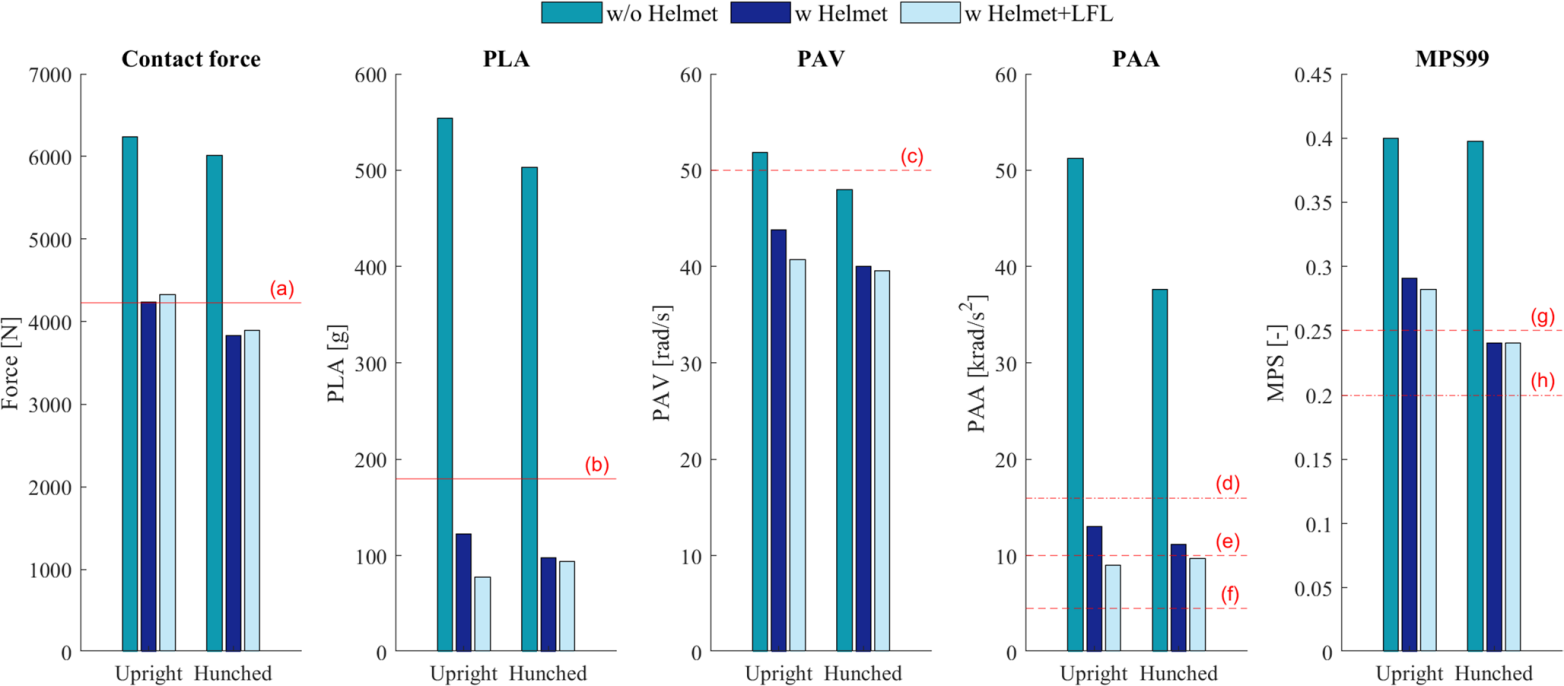
Head impact forces, peak linear accelerations (PLA), peak angular velocities (PAV), peak angular accelerations (PAA), and the 99th percentile maximum principal strain (MPS99) of the brain for the impact scenario deemed most plausible (Scenario C, −35° impact angle). Some reported thresholds are included in the plots, red line for skull fracture criteria, red dashed line for SDH criteria and red dash-dotted line for other criteria: (a) A lower limit of reported value for peak fracture forces of PMHS cranial bone, (b) Suggested threshold for a 5% risk of skull fracture, (c)(e)(f)(g) Suggested thresholds for SDH, (d) Suggested threshold for moderate to severe DAI, (g)(h) Suggested limit for DAI (the onset of malfunction neurons in the brain, which could be viewed as a first stage of DAI). See Table 1 for references

In Fig. 6, the peak contact force acting on the head, as well as the head center of gravity peak kinematics, are plotted. The kinematic pulses were extracted from the HBM and applied to ADAPT in order predict the MPS of the brain. The obtained MPS values are also included in the figure. Judging by these results, the helmet radically reduced most of the selected metrics, in particular the impact force and the head accelerations.

The LFL layer provided an additional, although small, reduction in PLA, PAV, PAA and MPS compared to the helmet without an LFL installed. The LFL was shown to reduce rotational kinematics and MPS to a larger extent in other impact scenarios which were included in the parametric study. The predicted injury metrics for other impact scenarios are included in Supplementary Appendix C.

**Fig. 7:**
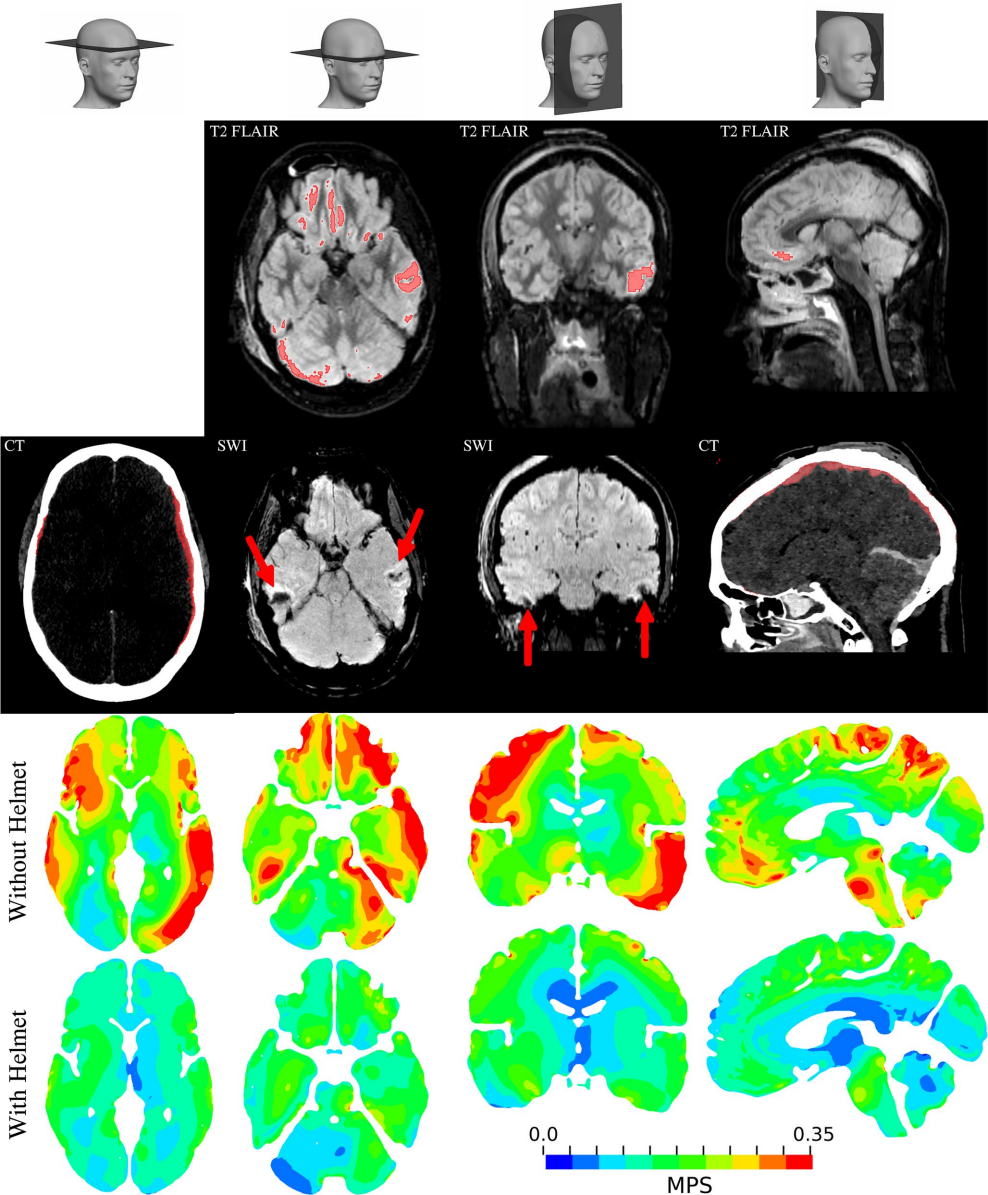
Lesions detected in the victim’s MRI and CT, along with corresponding sections of ADAPT model, showing the accumulated MPS distributions over the brain. The MPS are plotted for the reconstruction both with and without a helmet (without LFL)

The predicted head impact, regardless of body posture, led to wide-spread strain of the brain tissue of relatively large magnitude, with MPS99 values of approximately 0.40 without a worn helmet. With a worn helmet, the MPS99 stayed below 0.30, ranging between 0.29 − 0.24. The accumulated maximum brain strains, meaning the absolute MPS of each element over the whole time interval, is presented in Fig. 7. The same cross-sections of the brain from the victim’s medical images, showing detected brain lesions, are included in the figure. As illustrated, many of the areas of high brain strain overlap with the victim’s lesions. When wearing a helmet, the concentrated regions of high MPS were notably reduced and did generally not reach MPS higher than 0.20.

## Discussion

In this study, a subject-specific head model has been used to reconstruct a real-life traumatic head injury, shedding new light on the recent advances in FE modeling of human injuries. By following a thorough process of approximating and deriving unknown input parameters, the skull fracture was predicted with remarkable similarity to the real- world fracture. A good correlation between regions of brain lesions and regions of predicted increases in brain strain was found. Moreover, the predicted deformation of the scalp was localized in corresponding regions of extracranial swelling. The results highlight how wearing a construction helmet might have saved the victim from lethal or severe head injuries.

The results suggest that the victim would most likely not have sustained a skull fracture if he had been wearing a helmet at the time of the accident. Firstly, in the hypothetical scenario in which the victim would have been wearing a helmet, the subject-specific FE head model did not predict the occurrence of a fracture. Secondly, on the basis of literature covering PMHS experiments, the reconstruction of the workplace accident did not predict impact forces high enough for breaking skull bone. Third, and final, the acceleration of the head CoG was predicted to be significantly lower than the suggested 5%-probability of skull fracture. Without a helmet, the unprotected head would be exposed to impact forces and G-forces as high as 6,000 N and 500 g respectively (see Fig. 6), which is to be compared with wearing a helmet, where forces would be reduced to below 4,000 N and 100 g. These levels of impact forces and head acceleration have reportedly not been associated with skull fracture.

The predicted brain strains indicate that severe and life-threatening brain injuries might have been prevented by a construction helmet. Maximum strains in the brain have previously been shown to have good correlation with brain injury [51]. In this study, the predicted MPS in corresponding regions of the documented lesions were reduced from > 0.35 to < 0.20 in most regions. Considering how strains above 0.20 have been reported to cause brain white matter damage [39], brain cell death [40], and cerebral vasculature rupture [36–38] this is a strong indication that the deformation of the brain, when wearing a helmet, does not reach a level that is considered to be severely damaging. The helmet reduced the MPS to a magnitude more-often associated with mild traumatic brain injury, such as what has been found for concussions when using brain models with the same brain material model and shear stiffness as the currently used ADAPT model [43].

According to medical notes from victim’s MRI, there was no compelling suspicion of DAI injuries. Although the victim might not have lived long enough and with sufficient circulation to produce prominent secondary indications of DAI such as micro bleeding and swelling, the predictions do not indicate that the impact with a helmet had generated head movement above the suggested thresholds for DAI in terms of angular acceleration (16 krad/s^2^)[42]. The levels of predicted brain strains were generally close to or below the levels that would indicate the onset of axonal failure or similar [39–42]. Bear in mind that DAI is one of the most severe and lethal brain injuries [46, 47].

In regards to SDH, which is also considered to be one of the most lethal brain injury types [44, 45], the prediction of them is still a subject of controversy. The few tolerance thresholds of SDH reported in literature have been in terms of rotational movements (PAA and PAV). In the current reconstructed workplace accident, the PAV and PAA was reduced to levels close to, but not always below, these suggested thresholds when a helmet was worn. Yet, with some of these studies dating back all the way to 1974, there is an obvious lack of subsequent literature supporting the proposed thresholds. There is still no consensus regarding injury mechanisms of brain injuries, and the absence of mechanism-based injury risk functions for SDH, SAH or contusions emphasize this knowledge gap. Many recent studies have nevertheless been studying MPS in the context of brain injury prediction. Judging by the predicted brain MPS in this study, the strain levels without a helmet were predicted in agreement with such preceding literature. For instance, Yuan et al. [54] recently reconstructed a video-documented alpine skiing crash, where a head impact had resulted in severe brain injuries, including subdural hematoma. In the thoroughly reconstructed accident, Yuan et al. reported MPS values of 0.62–0.69 within volumes of edema and micro-bleedings. Furthermore, Fahlstedt et al. [52] performed in-depth reconstructions of three bicycle accidents, where a good correlation between predicted patterns of strains and areas of contusions seen in medical images was observed. The MPS of the corresponding areas of lesions were reportedly between 0.30 and 0.72. With these previous studies in mind, MPS levels below 0.20 in areas of detected lesions which were predicted in this study, might indicate that the risk of SDH/contusions/microbleedings are low.

Although the built-in rotational protection (LFL) was not shown to dramatically reduce the head injury risk metrics in Scenario C (𝛼 = −35°), other conclusions could be drawn based on the other impact scenarios included in the parametric study. In other evaluated impact scenarios (𝛼 of + 35° and 0°), the LFL was shown to notably decrease the MPS and rotational kinematics compared to the helmet without a built-in LFL. In Supplementary Appendix C, a wider selection of impact angles and scenarios (Scenario A, B, C) are presented. The LFL was shown to reduce MPS in all other impact scenarios compared to the helmet without the built-in LFL.

Despite the striking correlation between the CT findings and the FE prediction, there were slight discrepancies between the predicted fracture and the victim’s actual fracture. This could have several explanations. One explanation may lie in the individual biological variation. In reality, human scalp thickness, skull stiffness and head mass vary among individuals. The material models used in this study represent a general population due to the unavailability of data, such as the victim’s head mass or bone density. The individual variations can have an impact on the fracture prediction, possibly giving rise to artifacts and deviations. To increase the accuracy of the fracture prediction, tuning of some input parameters might have been needed in order to account for individual variation. The model’s sensitivity to these input data have been investigated in Supplementary Appendix B. It was shown how biological variation parameters and the impact velocity had an effect on the severity of the predicted skull fracture. Yet, the path of the dominating fracture line remains the same. Furthermore, the influence of the skull morphology, thickness and size have previously been claimed to be some of the most influential factors in skull fracture prediction [17, 24]. These are all factors that have been taken into account in this study.

Another reason for the differences in predicted and actual fracture may be that the ADAPT model lacks some structures of the skull, especially regarding the facial bones. The victim’s fracture ran across structures that the ADAPT partially lacks, including the sphenoidal sinus. These sections of the fracture could therefore not be predicted exactly, although the model correctly predicted how the fracture propagated in the same direction towards the orbital roof. A third reason may be that the sutures are modeled with the same material model as the surrounding cranial bone in the ADAPT model, something that can also possibly have an impact on the fracture propagation in this case, since the victim sustained suture widening. Yet, research indicates that the sutures do not differ significantly in bending stiffness and strength as comparable layered cranial bone structures [53]. The mentioned variations are not believed to have a major impact on the kinematics, brain strains or contact forces presented in this report. 

The case that has been investigated in this study consists of many unknowns and uncertainties: How and where was the victim standing at the moment of impact? Where did the jack post strike the victim? With reference to the results of the sensitivity study (Supplementary Appendix B), the answers to these questions could influence the prediction results. Knowing the answers would increase not only the accuracy of the results, but also the credibility of the drawn conclusions. However, this study demonstrated how estimations of unknown parameters can be made by employing thorough, scientific and case-specific methods to still achieve adequate predictions of human injury. Following such methods, FE reconstructions can be used as supporting biomechanical evidence to facilitate the objectification of forensic evaluations.

## Conclusions

In this study, a real-world workplace accident has been reconstructed utilizing state-of-the-art FE models and methodologies. The study has been used to provide the police with biomechanical evidence to support an ongoing liability investigation. As a result, the following conclusions can be drawn based on the presented material:

- According to the biomechanical reconstruction presented in this study, a free falling jack post of 5 m and 35 kg could have caused the victim to sustain a severe linear skull fracture running along the left parietal bone across the skull base towards the left inner facial bones.
- The results suggest that the victim would have been spared a skull fracture if he had been wearing a helmet at the time of the accident.
- The results indicate that the victim would have sustained milder and less life-threatening brain injuries if he had been wearing a helmet.

This case study serves as a showcase of how FE accident reconstruction methods can be used as a complementary tool in forensic and police investigations. It also conveys an important message: construction helmets can prevent lethal head injuries and are imperative to combat the globally high incidence of fatal work-related accidents.

## Transparency, rigor, and reproducibility statement

This study presents a computational reconstruction of a real-world head trauma case using finite element (FE) simulation. All modeling assumptions, boundary conditions, and material properties are described in detail in the Methods section to ensure clarity and scientific rigor. The FE model was benchmarked against published experimental data where applicable. No human or animal subjects were directly involved, and no clinical trials were conducted. Due to intellectual property restrictions and the need to preserve subject anonymity, the simulation files and underlying data are not publicly available.

## Supplementary Information

Below is the link to the electronic supplementary material.

Supplementary Material 1 (PDF 2.44 MB)
