# Supplementary material for: Can construction helmets save lives? Evidence from a biomechanical reconstruction of a work-related head trauma
Source: Int J Legal Med. 2026 Jan 6;140(3):1849–62. doi: 10.1007/s00414-025-03695-9 (PMC13161303; doi:10.1007/s00414-025-03695-9)
Supplement: Supplementary file 1 — Supplementary Material 1 (PDF 2.44 MB) [file 414_2025_3695_MOESM1_ESM.pdf]

Appendix A: Helmet validation

The helmet model was based on the design and performance of the *Guardio Armet helmet*, se Figure 1.

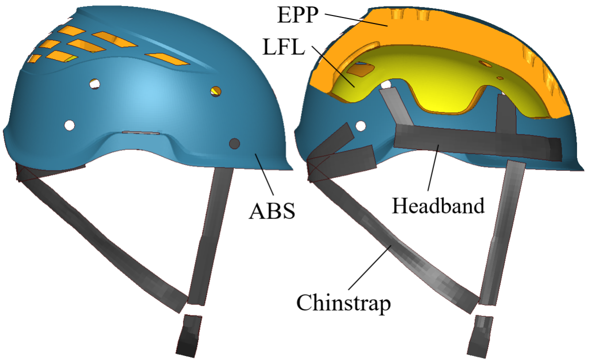

Figure 1. The helmet model, representing a construction helmet currently available in the Swedish market.

The model was compared to test results from a set-up similar to the certification test standard of EN 397 for industrial safety helmets. The tests consisted of a 5 kg hemispherical striker with the radius 50 mm, impacting the crown of the helmet when positioned on a rigid metal EN960 headform. The resulting force was measured in the base of the headform. The helmet was tested in two impact velocities corresponding to the drop heights of 1 m and 2 m, in ambient conditions. In the simulation the force was measured in a node set in the base of the headform.

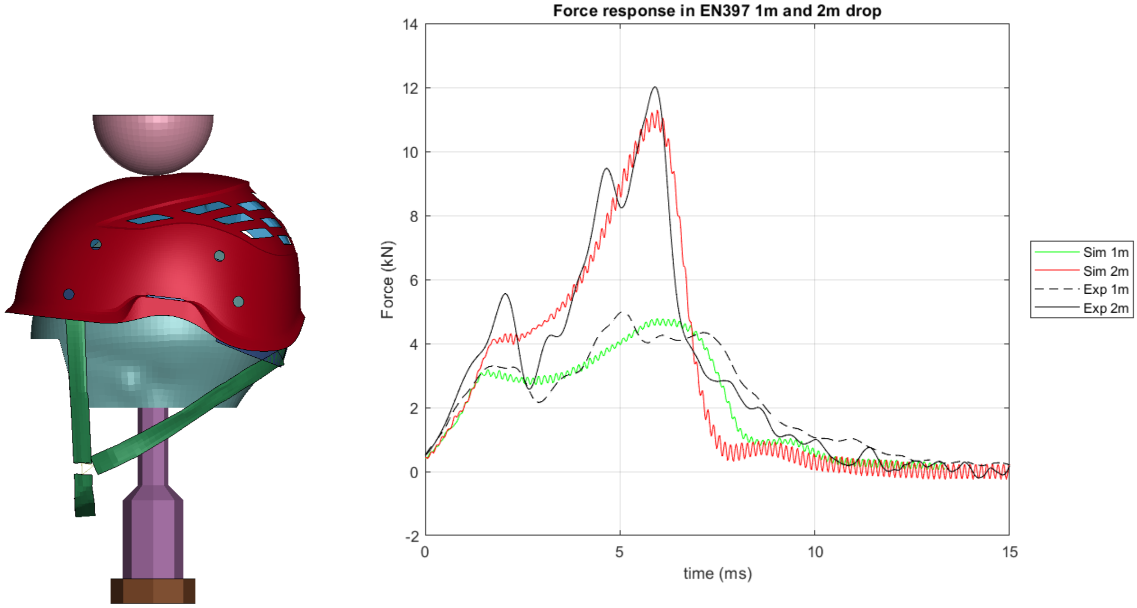

Figure 2. Test set-up according to EN397 and resulting force at impact for a 1m and a 2m drop, respectively.

Appendix B: Extended results

The three impact scenarios (A, B and C), were reconstructed using the ADAPT head model, impacting the HBM straight from the right, i.e. impact angle $\beta = 0°$. A skull fracture was predicted by the model in all three scenarios. As illustrated in Figure 3, Scenario A and B resulted in severe skull fractures, where the model predicted a depressed and comminuted fracture. In Scenario C, a linear skull fracture was predicted by the model, following a path from the impact point towards the frontal lobe. Due to the severity of the fractures predicted in Scenario A and B, these two scenarios were omitted as non-plausible while Scenario C, with an incline angle $\alpha = 54°$ and angular velocity of $\omega = 1.5$ rad/s ($v_{res} \approx 4.6$ m/s), was evaluated further.

From the CT segmentation, a swelling was observed near the assumed region of impact. It was found that these regions of swelling aligned well with the pattern of scalp deformation predicted by the ADAPT model in the most plausible scenario (Scenario C, α=-35°). The scalp's deformation during the simulated impact is presented in Figure 4, along with a thickness map of the scalp, which was generated from the segmentation of the victim's CT. Note that the victim was laying down during

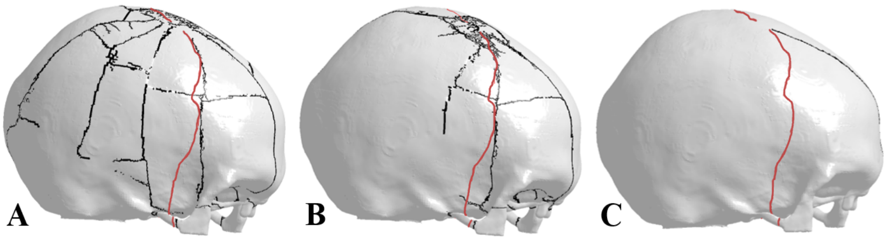

Figure 3. Predicted fractures in Scenarios A, B and C. The black elements represent eroded elements, i.e. fracture. The red line represents the fracture seen in the victim's medical images.

the scanning, allowing the scalp to deform under the weight of the head, potentially explaining the swelling in the occipital region. Furthermore, accumulated fluid can trickle down under the influence of gravity.

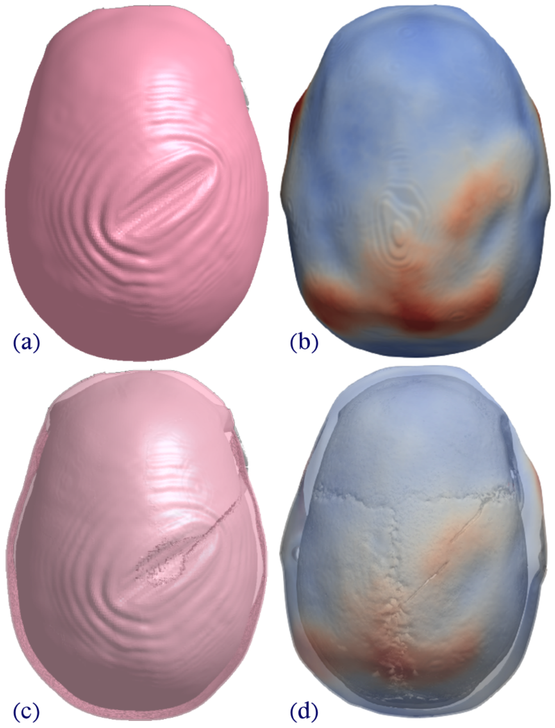

Figure 4. Top views of: (a) The ADAPT head model mid-impact showing the predicted scalp deformation as it is being indented by the falling jack post. (b) Thickness map of the scalp, generated based on the segmentation of the victim's CT. Red regions represent larger distance between the skull outer surface and the scalp outer surface, indicating regions of swelling. (c) Transparent view of the scalp showing the underlying predicted fracture. (d) Transparent view of the thickness map showing the underlying segmented fracture.

To increase the accuracy of the fracture prediction, tuning of some input parameters might be needed in order to to account for individual variation. However, some of these parameters were not available, such as the victim's head mass or cortical bone strength. To study the model's sensitivity to some of these input data, a selection of the variables were altered, see Figure 5. In the figure, it is shown how biological variation and the impact velocity has an effect on the severity of the predicted skull fracture. However, the path of the dominating fracture line remains the same.

Appendix C: Other impact scenarios evaluated using THUMS

In Table 2, the impact kinematics and metrics of a range of impacts are presented.

Note, that in some cases, the helmet changes the conditions of the impact and thus increases the risk of injury. When the jack post has a higher impact velocity, as in scenarios A and B, the helmet seemingly increases the brain strain markedly. In scenario A, the maximum brain strain goes from 0.32 without a helmet to 0.54 with a helmet. Note that the message in this case is not that helmets can cause more damage at high impact velocities. What happens is that the helmet in some scenarios influence the characteristics of the impact. Without a helmet, the jack post slides off the head in scenarios A and B. With the helmet on the head, more space is taken up under the falling jack post and the head is pulled down by the weight of the post. This contributes to contact loads on the head for a longer period of time and thus generates greater strain in the brain,

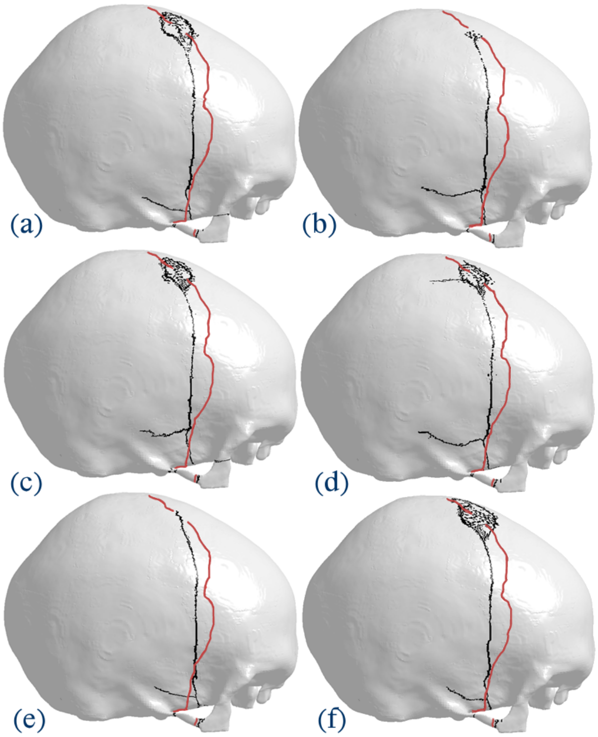

Figure 5. Predicted skull fractures illustrating the sensitivity of the model to biological variation. The black elements represent eroded elements, i.e. fracture. The red line represents the fracture seen in the victim's medical images. View (a) shows the original predicted fracture, while the other shows the predicted fracture with alterations to the input parameters: (b) no facial mass, (c) 1 mm thinner scalp, (d) Lower tensile strength of cortical bone, (e) 0.5 m/s lower impact velocity and (f) 0.5 m/s higher impact velocity.

see Figure 6. In these cases, however, the helmet still reduce the risk of fracture, as the impact force is still reduced. Bear in mind, that scenarios A and B are probably not what caused the victim's injuries, as only scenario C with an impact angle of -35° predicts a location and extent of the skull fracture that is consistent with the plaintiff's skull fracture.

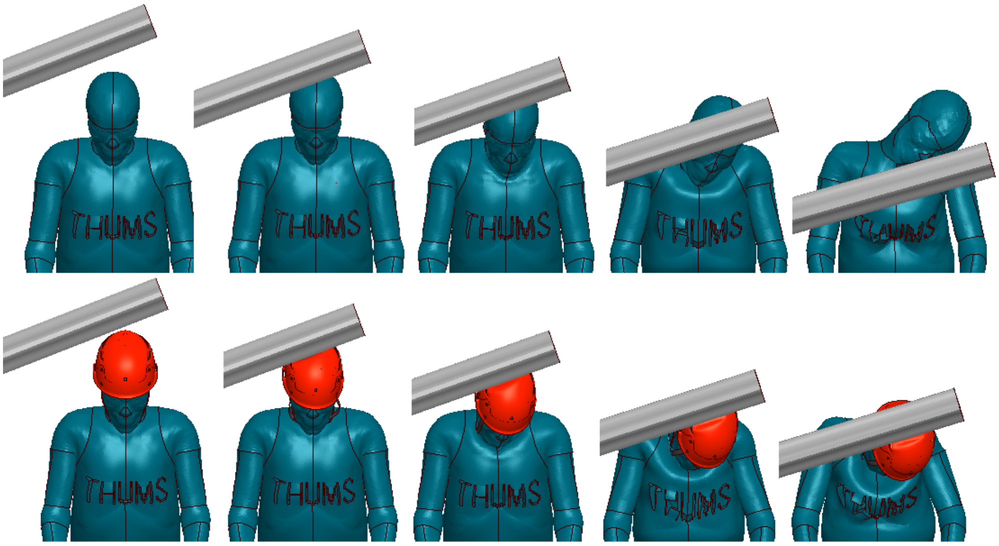

Figure 6. HBM simulations plotted for Scenario A, without and with a helmet, in intervalls of 10 ms (0° impact angle).

Tab. 2. Impact data of several impact scenarios. Note that the MPS are extracted from the KTH head model, not ADAPT.

Scenario	β	Posture	Helmet	MPS	PLA	PAV	PAA	F
A	0°	Hunched	No Helmet	0.32	511	36.6	40.9	11 800
			Helmet	0.54	144	45.1	15.0	8 550
			Helmet+LFL	0.28	132	37.7	5.99	7 470
B	0°	Hunched	No Helmet	0.28	383	40.0	27.8	8 970
			Helmet	0.41	110	37.6	11.5	6 470
			Helmet+LFL	0.23	109	28.2	6.20	5 970
C	0°	Hunched	No Helmet	0.25	480	31.5	49.7	5 800
			Helmet	0.26	63.7	27.3	5.92	3 730
			Helmet+LFL	0.16	64.0	21.3	3.63	3 700
C	0°	Upright	No Helmet	0.31	599	34.9	62.7	5 390
			Helmet	0.27	115	36.6	9.40	4 620
			Helmet+LFL	0.23	77.5	33.8	4.63	4 710
C	+35°	Hunched	No Helmet	0.35	248	27.3	17.2	6 510
			Helmet	0.29	58.1	28.2	5.70	3 160
			Helmet+LFL	0.22	56.4	26.3	4.01	2 990
C	+35°	Upright	No Helmet	0.29	664	33.6	55.7	5 880
			Helmet	0.16	78.3	25.6	4.04	4 780
			Helmet+LFL	0.13	78.5	26.2	4.74	4 770

Appendix D: Methodological choices

In this Appendix section, we motivate why and when we use an HBM versus a head-only model.

First, the victim's skull fracture was predicted using a subject-specific head model, modeled with a mesh fine enough to achieve a high-resolution prediction of the propagation and pattern of a skull fracture[17]. Since skull fractures are mainly caused by applied contact loadings that are large enough to cause the skull to break[19], the victim's skull fracture was presumed to have occurred primary to the head impact, nearly momentaneously to the blow to the head. Thus, the skull fracture was studied in a time scale of <15 ms, capturing solely the contact phase of the striking object. Second, the victim's sustained brain injuries were studied using a whole-body model, modeled with a coarser mesh to allow for a longer simulation time. Contrary to skull fractures, brain injuries can also occur because of indirect loading, for example through rapid rotational motion of the head. A rapid motion of the head can lead to relative movement of the brain within the skull, causing bridging veins to strain and possibly rupture[19].

Consequently, the victim's brain injuries were presumed to have occurred due to the skull fracture itself, and/or occurred secondary to head impact, caused by the rapid post-impact movement of the head. Therefore, the brain injuries were studied in a time scale of <150 ms. Within this time frame, the body would have time to respond to the heavy head blow, which would have an influence on the head kinematics. Due to this, a whole-body model was used to predict the head kinematic response, as a head model alone would be insufficient.
